# Novel Advances in Cell-Free Therapy for Premature Ovarian Failure (POF): A Comprehensive Review

**DOI:** 10.34172/apb.2024.059

**Published:** 2024-07-31

**Authors:** Yahya Yahyavi, Niloufar Kheradi, Abbas Karimi, Abbas Ebrahimi-Kalan, Fatemeh Ramezani, Soudabe Yousefi, Shirin Teymouri Nobari, Hourieh Sadrekarimi, Mohammad Nouri, Mahdi Edalati

**Affiliations:** ^1^Department of Molecular Medicine, Faculty of Advanced Medical Sciences, Tabriz University of Medical Sciences, Tabriz, Iran.; ^2^Molecular Medicine Research Center, Tabriz University of Medical Sciences, Tabriz, Iran.; ^3^Department of Neurosciences and Cognition, Faculty of Advanced Medical Sciences, Tabriz University of Medical Sciences, Tabriz, Iran.; ^4^Department of Reproductive Biology, Faculty of Advanced Medical Sciences, Tabriz University of Medical Sciences, Tabriz, Iran.; ^5^Department of Clinical Biochemistry, Faculty of Medicine, Urmia University of Medical Sciences, Urmia, Iran.; ^6^Department of Immunology, Faculty of Medicine, Tabriz University of Medical Science, Tabriz, Iran.; ^7^Department of Laboratory Science, Faculty of Paramedicine, Tabriz University of Medical Sciences, Tabriz, Iran.

**Keywords:** PRP, Exosome therapy, In vitro activation, Stem cell therapy, Mitochondrial targeting therapies, Theca cells signaling pathways, PLT-Exos

## Abstract

Premature ovarian failure (POF), is a condition characterized by the early decline of ovulation function. POF is a complex disorder that can be caused by various factors, and the idiopathic form represents a significant proportion of POF patients. Hormone replacement therapy (HRT) is currently considered the first-line treatment for POF. This review aims to provide a comprehensive overview of recent advancements in platelet-rich plasma (PRP), in vitro activation (IVA), stem cell therapy, exosome therapy, microRNAs, and mitochondrial targeting therapies as a promising cell-free therapeutic approach in reproductive medicine. PLT-Exos, a new generation of cells, has been used to treat POF for more than a decade and has been shown to attenuate oocyte morphology and promote the differentiation of theca cells through the upregulation of PI3K/Akt and Bcl2, as well as the downregulation of the Smad and Bax signaling pathways. This review summarizes the current state of the art in the field of PLT-Exos and discusses the advantages and limitations of their potential clinical applications.

## Introduction

 Premature ovarian failure (POF), is a condition characterized by the early decline of ovarian function. Women with POF may experience symptoms similar to menopause. While the average age of menopause in the US is 51 years, POF affects women under the age of 40, leading to a cessation of ovulation and a decline in hormonal ovarian functions.^1,2^ Based on worldwide epidemiological statistics, POF impacts about 1% of females under 40 years old, 0.1% of females under 30, and 0.01% of females under 20.^3^ Typical symptoms consist of absence of menstruation, inability to conceive, night sweats, hot flashes, and urogenital issues. POF is a multifaceted condition that can result from a variety of factors, including genetic influences (like Turner’s syndrome, fragile X messenger ribonucleoprotein1, premutation, galactosemia), autoimmune disorders, mitochondrial irregularities, medical treatments (such as chemotherapy, radiotherapy, and surgeries), infectious illnesses (like tuberculosis, mumps, malaria, varicella, shigella, cytomegalovirus, and herpes simplex), and environmental elements.^4^ Moreover, a notable number of individuals with POF experience an idiopathic version of the disorder with an unknown cause.^5^ Currently, hormone replacement therapy (HRT) is the first-line treatment for POF. Long-term HRT treatment can help control symptoms by replacing estrogens in the body and preventing the adverse consequences of POF.^6^ Nonetheless, HRT is unable to fully revive ovarian function, and prolonged utilization may lead to negative outcomes such as cardiovascular issues, osteoporosis, and breast cancer.^7^ In vitro activation (IVA) presents a different method for addressing POF. This therapy has the potential to boost the quantity of developed eggs in POF individuals, enabling them to achieve successful pregnancies. Nevertheless, the effectiveness of IVA may be limited when the quality of oocytes related to age has deteriorated.^8^ Researchers are developing new strategies, including mitochondrial activation, stem cell therapy, intra-ovarian infusion of platelet-rich plasma (PRP) and exosomes transportation, to address POF. These emerging treatments aim to provide additional options for POF patients.^5^ In recent decades, PRP therapies have gained significant attention in the field of regenerative medicine. Numerous systematic reviews and meta-analyses have documented the promising outcomes of PRP therapies in various clinical areas, such as orthopedic surgery, plastic surgery, dermatology, gynecology, cardiac surgery, maxillofacial surgery, pain management, spinal disorders, sports medicine, and more.^9^ The main reason PRP is beneficial for healing comes from the biomolecules released by platelets, particularly growth factors. Platelets possess the capability to release growth factors, cytokines, and substances that regulate the extracellular matrix, aiding in the process of vascular regeneration, tissue healing, and stimulation of stem cells.^10^ Although the scientific rationales behind PRP’s effects are not entirely comprehended, recent advancements in platelet research have shed light on the subject. Specifically, the focus has been on Extracellular vesicles (EVs) released by platelets, such as exosomes, which have gained significant attention due to their functionality. In the past few years, research on these vesicles has increased exponentially.^11^ Platelet-derived exosomes (PLT-Exos) are a specific type of EVs comprising small lipid bilayer vesicles released by platelets. These PLT-Exos make up more than 75% of EVs present in the blood. Ranging in size from 30 to 100 nm, they are formed from the nucleosome and are released through the merging of multivesicular bodies (MVBs) with the cell membrane.^12^ Following their release, exosomes have the capability to convey a range of materials to recipient cells, such as mRNAs, microRNAs (miRNAs), proteins, lipids, and molecules like ceramide and phosphatidylserine.^13^ Platelets, which are derived from megakaryocytes in the bone marrow, play a critical role in blood function and are engaged in a number of physiological and pathological processes such as hemostasis, thrombosis, immune response and wound healing. When platelets are activated, they release PLT-Exos that facilitate communication between cells by delivering microRNAs and proteins.^14^ The use of PLT-Exos derived from one’s own PRP has demonstrated beneficial impacts on ovarian function. It assists in restoring the estrous cycle, elevating hormone and anti-Müllerian hormone (AMH) levels, as well as boosting follicle count. Additionally, it prevents follicular degeneration, improves fertility rates, and reduces the time required for conception.^15^ The most important mechanism behind rejuvenation of ovarian induced by PLT-Exos involves the regulation of granulosa cells. This leads to increased proliferation and decreased apoptosis, with the involvement of specific signaling pathways like PI3K/Akt and BAX.^16^ PLT-Exos also carry various microRNAs, including miR-144-5p, miR-126-3p, and miR-369-3p, which inhibit granulosa cell apoptosis through different pathways. Additionally, PLT-Exos help attenuate fibrosis of ovarian tissue and promote theca cells differentiation by inhibiting the TGF-β1/Smad3 signaling pathway.^17^ These effects contribute to the reconditioning of ovarian function in cases of POF. The purpose of this review is to provide a comprehensive overview of the recent advancements in treatment of POF patient and uses of PLT-Exos as a promising cell-free therapeutic approach in reproductive medicine. The review aims to elucidate the underlying molecular mechanisms of PLT-Exos, as well as discuss the advantages and limitations of their potential clinical applications. Based on current research, it is anticipated that PRP-derived products, such as PLT-Exos or PRP hydrogel, will emerge as more effective and safer alternatives to traditional PRP in the field of tissue repair and regeneration.

## Pathophysiology of POF

 One percent of women are affected by POF, and most instances are idiopathic. The causes of POF are varied and may include chromosomal and genetic abnormalities, autoimmune ovarian destruction, environmental factors and iatrogenic factors. The clinical presentation of POF is variable and may include symptoms of estrogen deficiency, infertility, menstrual disturbance, or syndromic conditions. The presence of increased blood follicle-stimulating hormone (FSH) concentrations ( > 40 IU/L) on at least two occasions spaced a few weeks apart is the basis for the diagnosis. This is necessary to ensure certainty as the diagnosis can be devastating and the natural history of POF can be unpredictable, with many women experiencing relapse and remission. Some women may also experience “fluctuating ovarian function”.^18^ In clinical settings, women diagnosed with POF exhibit a pregnancy probability of approximately 1%-5%. Given this inherent rate of fertility, any unverified claims of successful POF management should be considered cautiously. Nonetheless, it is crucial to inform women with POF about their condition to ensure they can make informed decisions regarding the use of contraception when necessary. The objective of secondary research in this context is to ascertain the underlying causes of POF and to track any associated complications.^19^ Ovarian biopsies are of limited value in research due to the diminutive and unrepresentative nature of the samples collected. In a similar vein, pelvic ultrasound does not offer predictive insights but is instrumental in identifying individuals who may be eligible for future oocyte preservation or maturation interventions. Ultrasonography, owing to its non-invasive character, fulfills a psychological role by aiding individuals in understanding and coming to terms with their condition, often marked by small ovaries and reduced follicular activity. The screening for autoimmune thyroid and adrenal antibodies stands as a vital subsequent test for tracking potential insufficiencies in thyroid and adrenal function, or vitamin B12 levels, especially when initial tests are inconclusive. Gathering a thorough family history can uncover the involvement of up to 30% of other female relatives, thereby facilitating the provision of genetic counseling.^20^ Genetic assessments are increasingly utilized in familial cases of POF and are also applicable to sporadic cases, offering a more cost-effective and efficient approach. For example, screening for pre-mutations in the FRAXA gene is common, with a detection rate of 15% in individuals with a confirmed family history and 3% in sporadic cases. Currently, karyotype analysis and FRAXA premutation screening are the only broadly available tests. These should be considered for individuals with a family history or those experiencing an unusually early onset of POF.^21^

## Aetiology of POF

 POF can be attributed to various causes including chromosomal abnormalities, genetic factors, autoimmune disorders, metabolic conditions (such as galactosemia), infectious diseases (like mumps), and iatrogenic factors (such as anticancer treatments). However, despite advancements in diagnostics, a significant number of POF cases still have no identifiable cause and are considered idiopathic (Table 1).^22^

**Table 1:** Etiology of POF

**POF causes**	**Example**	**References**
Genetic	Turner’s syndrome and X chromosome defects, Down’s syndrome, premutation of the fragile X messenger ribonucleoprotein 1 (FMR1) gene	^23-29^
Autosomal disorders (enzyme deficiencies)	Galactosemia, 17α-hydroxylase, aromatase	^30-32^
Autoimmune	Adrenal disease, thyroid complications, diabetes mellitus, vitiligo, myasthenia gravis, Addison’s disease, systematic lupus erythematosus, celiac disease, autoimmune polyendocrine syndrome	^33-37^
Mitochondrial abnormality	Absence of protein Mitofusin 2 (MFN2), mutations of dynamin-related protein 1 (Drp1), absence of mitochondrial proteases	^38-46^
Infections	Viral (mumps, cytomegalovirus, herpes and varicella), bacterial (tuberculosis and shigella), parasite (malaria)	^47-49^
Iatrogenic factors	Chemotherapy, radiotherapy, pelvic surgery	^50-55^
Environmental toxin	Cigarette smoking, heavy metal, solvent, pesticides, plastics, industrial chemicals	^56-58^

## Genetic reasons of POF

###  Turner’s syndrome and X chromosome defects

 Failures involving the X chromosome, such as Turner’s syndrome and X chromosome abnormalities, have been associated with POF. These anomalies may involve the entire loss of an X chromosome (Turner syndrome), or minor problems such as autosome X translocations or deletions. The precise genetic locus causing POF is still unclear, though. In cases of Turner syndrome variants, individuals with a mosaic 45, X/46, XX karyotype may not exhibit the representative phenotypic property of the syndrome but can still present with POF. X chromosome deletions have been observed in two specific regions: POF1 at Xq21.3-q27 and POF2 Xq13.3–Xq21.1.^23,24^ Interruption of several genes involved in ovary development and oogenesis within these critical regions may occur due to balanced translocations, leading to POF. It is significant to remember that not every X chromosomal breakpoint is connected to POF.^25^

###  Down’s syndrome

 Genetic assessments are increasingly utilized in familial cases of POF and are also applicable to sporadic cases, offering a more cost-effective and efficient approach. For example, screening for pre-mutations in the FRAXA gene is common, with a detection rate of 15% in individuals with a confirmed family history and 3% in sporadic cases. Currently, karyotype analysis and FRAXA premutation screening are the only broadly available tests. These should be considered for individuals with a family history or those experiencing an unusually early onset of POF.^26^

###  Premutation of the fragile X messenger ribonucleoprotein 1 (FMR1) gene

 The expansion of CGG trinucleotide repeats (ranging from 55 to 200 CGGs) within the 5’ untranslated region is indicative of a premutation in the FMR1 gene. This genetic alteration results in elevated levels of FMR1 mRNA.^27^ The premutation of the FMR1 gene is associated with a spectrum of disorders known as fragile X-premutation-associated conditions (FXPAC). The underlying molecular mechanisms of FXPAC include the formation of co-transcriptional R-loops, the toxicity of FMR1 mRNA stemming from its aggregation into nuclear foci, the sequestration of various CGG-repeat-binding proteins, and the initiation of repeat-associated non-AUG (RAN) translation, which may produce deleterious proteins. These molecular disturbances can lead to significant cellular consequences, such as compromised mitochondrial function and neuronal loss.^28^ Individuals with the FMR1 gene premutation may exhibit a range of symptoms and characteristics, all of which are categorized within the spectrum of FXPAC. FXPAC includes disorders such as fragile X-associated tremor/ataxia syndrome (FXTAS), fragile X-associated primary ovarian insufficiency (FXPOI), and fragile X-associated neuropsychiatric disorders (FXAND). A thorough understanding of both the molecular and clinical aspects of the FMR1 gene premutation is crucial for accurate diagnosis, informed genetic counseling, and the provision of tailored care to affected individuals and their families.^29^

## Autosomal disorder (enzyme deficiencies)

 Galactosemia, a rare autosomal recessive disorder, is caused by a deficiency in the enzyme galactose-1-phosphate uridyltransferase (GALT), which is located on the 9p13 chromosome. Female individuals with galactosemia often exhibit a significant occurrence of POF, with rates ranging between 60% to 70%. This disorder is marked by the harmful impact of galactose or its byproducts on follicular structures.^30^ During fetal development, the presence of galactose is known to reduce the initial population of oogonia, the precursor cells to eggs. This reduction hastens follicular atresia, the process by which ovarian follicles degenerate and are lost. The detrimental effects of galactose are implicated in the premature depletion of ovarian follicles, potentially leading to infertility or early onset menopause in those affected.^31^ Malfunctions in proteins and enzymes crucial for the steroidogenesis process, like shortages in aromatase and 17α-hydroxylase, can contribute to POF. Cells responsible for steroid production (such as hilar cells, granulosa cells, theca interna, and corpus luteum) and autoantibodies targeting these cells are commonly observed in POF cases associated with Addison’s disease.^32^

## Autoimmune causes of POF

 Autoimmune mechanisms may play a role in up to 30% of POF cases. The evidence supporting an autoimmune cause includes: 1. Presence of lymphocytic oophoritis: Oophoritis is characterized by the infiltration of immune cells such as macrophages, natural killer cells, T lymphocytes, plasma cells, and B lymphocytes into the ovaries. 2. Demonstration of ovarian autoantibodies: Anti-ovarian antibodies have been detected in individuals with POF, indicating an immune response against ovarian tissue. 3. Associated with autoimmune disorders: diabetes mellitus, thyroid issues, and adrenal illness.^33^ Autoimmune polyendocrine syndromes (APS) types I and II are recognized as additional etiologies of ovarian failure. Type I APS is typified by a constellation of conditions including POF, chronic mucocutaneous candidiasis, hypothyroidism, and adrenal insufficiency. Conversely, Type II APS is characterized by the co-occurrence of type 1 diabetes, POF, adrenal insufficiency, and thyroid autoimmune disorders. Additionally, a spectrum of autoimmune disorders such as myasthenia gravis, Adison’s disease, systemic lupus erythematosus, vitiligo, celiac disease, among others, have been linked to the onset of POF.^34^ In clinical practice, the primary method for identifying an autoimmune etiology in the evaluation of POF is to screen for the presence of coexisting autoimmune diseases.^35^ Most women with POF, according to research, tested positive for at least one organ-specific autoantibody. Anti-thyroid antibodies are the most often discovered autoantibodies, accounting for 20% of cases. Subclinical involvement of non-ovarian autoimmune disease may exist in some cases.^36,37^

## Mitochondrial abnormalities

 Mitochondria are responsible for generating energy in cells and are unique in that they have their own genetic material called mitochondrial DNA (mtDNA). Their main function is to create ATP through oxidative phosphorylation. Additionally, mitochondria play a crucial role in regulating various cellular processes including apoptosis, redox potential, and calcium signaling.^38^ They are the main source of reactive oxygen species (ROS) in cells. The most significant relationship between mitochondria and oocyte quality and embryonic development is this one. Several factors, such as malfunctioning mtDNA, heightened oxidative harm, changes in membrane potential, and reduced production or removal of mitochondria, play a role in the aging of ovaries due to mitochondrial issues.^39^ Reduced mtDNA content, strand breakage, point mutations, and oxidative damage are all considered forms of mtDNA malfunction. According to studies, the mtDNA content of people with POF is substantially lower than that of healthy fertile women. Furthermore, because protective histones and DNA repair enzymes are absent from mtDNA, it is prone to mutations.^40^ Telomere shortening can result from significant effects on reactive ROS production and mitochondrial proteostasis, even from a single-point mutation in the mtDNA. ROS levels are frequently noticeably higher in POF populations.^41^ An overabundance of ROS leads to mtDNA mutations and energy deficit, which accelerate aging. Further enhancing the generation of ROS, mtDNA mutations set off a damaging cycle that can lead to cell death.^42^ Furthermore, cellular antioxidant defenses may be overwhelmed by an excess of ROS, which can result in oxidative stress (OS) and early aging.^43^ Oocyte aging is also impacted by disruptions in mitochondrial dynamics, such as aberrant fusion, changes in mitochondrial metabolism, and anomalies in calcium homeostasis.^44^ The protein Mitofusin 2 (MFN2) plays a key role in mitochondrial fusion, and the absence of MFN2 in oocytes contributes to female infertility.^45^ Another important factor for maintaining oocyte quality is dynamin-related protein 1 (Drp1), which is involved in mitochondrial fission. Drp1 deletion has been linked to ovulation problems and follicular dysplasia, according to studies. Moreover, the lack of mitochondrial proteases might accelerate the aging process of oocytes and cause illnesses linked to the mitochondria.^46^

## Infectious causes of POF

 Viral oophoritis, particularly mumps oophoritis, is recognized as a potential cause of idiopathic POF. Other infections, including tuberculosis, malaria, varicella, shigella, cytomegalovirus, and herpes simplex, have been implicated in the development of POF. It has been observed that ovarian failure occurs in 2%-8% of women affected by mumps oophoritis, although this condition is typically transient.^47-49^

## Iatrogenic factors

 Radiation and chemotherapy can cause POF in people receiving treatment for malignant illnesses. Radiation therapy field, age, and dose all affect the effects of radiation therapy. For women under 40, a dose of 20 Gy may result in complete ovarian failure; for older women, a dose of only 6 Gy may do so. Radiation and chemotherapy have very little gonadotoxic effect on the prepubertal ovary. The likelihood of experiencing premature menopause is low in women who undergo radiation treatment that does not target the pelvic area. Sixty to one hundred percent of patients can maintain ovarian function after ovary relocation (Ovariopexy).^50-52^ A substantial risk factor for POF is cytotoxic chemotherapy administered to young women for a variety of malignant disorders, especially when combined with alkylating drugs, which raise the risk by a factor of nine. The risk of POF is four times higher in teenagers undergoing chemotherapy, and it rises by a factor of 27 in women between the ages of 21 and 25. Although practically all pelvic surgeries carry some risk of ovarian injury due to disruption of the ovary’s blood supply or inflammation, this risk is negligible for most routine procedures. POF may also result from other pelvic interventional procedures such uterine artery embolization, which damage the ovary’s blood supply.^53-55^

## Environmental toxins

 Research has extensively examined the impact of cigarette smoking as a toxin that can influence ovarian function. Studies show that, on average, women who smoke tend to reach menopause at an earlier age compared to nonsmokers, indicating a potential harmful influence of cigarette smoking on ovarian function.^56^ Chang and colleagues found a higher likelihood of idiopathic POF linked to cigarette smoking.^57^ There have been reports of women with epilepsy having a higher chance of experiencing POF. Nevertheless, the existing information on the impact of endocrine disruptors, heavy metals, solvents, pesticides, plastics, industrial chemicals, and cigarette smoke on female reproductive health is not definitive. Seeking advice from a healthcare professional is crucial for accurate diagnosis and treatment.^58^

## Treatment strategies

 HRT does not restore ovarian function, but it is thought to be a physiological substitute for progesterone and estrogen. IVA, mitochondrial activation, stem cell and exosome therapy, and intraovarian infusion of PRP are some of the novel approaches that are presently being investigated. These treatments must first demonstrate their safety and effectiveness in order to be approved for use in clinical settings; they are currently in the experimental stages. Among these approaches, stem cell transplantation, PRP therapy, and the transportation of their exosomes have shown promising results as the most effective methods^59^ (Table 2).

**Table 2:** Summarizing the different therapies and techniques

**Therapy/Technique**	**Description**	**Reference**
Hormone replacement therapy (HRT)	Physiological replacement of ovarian steroid hormones until natural menopause	^60-64^
In vitro activation (IVA)	Disrupting the Hippo signaling pathway to stimulate dormant primordial follicles	^65-69^
Mitochondrial activation	Using pharmacological agents like CoQ10, resveratrol, melatonin, and rapamycin to restore mitochondrial function	^70-73^
Mitochondrial transfer	Novel techniques like pronuclear transfer, spindle transfer, and germline mitochondrial energy transfer to replace defective mitochondria	^74-84^
Stem cell therapy	Using mesenchymal stem cells (e.g., from bone marrow, adipose tissue, menstrual blood, umbilical cord) to regenerate ovarian function	^85-94^
Platelet-rich plasma (PRP) therapy	Intraovarian injection of autologous PRP to stimulate follicular growth and ovarian rejuvenation	^95-105^
Exosome therapy	Using extracellular vesicles (exosomes) derived from various stem cell sources to modulate ovarian function	^106-120^

## Hormone replacement therapy

 Typically, it is common practice to provide physiological replacement of ovarian steroid hormones until the age of natural menopause around 50. The approach to HRT in younger women varies slightly from that in older women, with the primary objective being to enhance overall quality of life.^60^ Young women may require a higher estrogen dose, and considerations for sexual function may necessitate vaginal estrogen and androgen replacement. Conjugated equine estrogen and 17 beta-estradiol have consistent effects on hot flashes.^61^ Transdermal estrogen offers rapid action and may reduce the risk of thrombosis. Subcutaneous estrogen replacement involves placing estradiol pellets, and testosterone implants may be included if indicated. Topical vaginal estrogen in various forms can be effective, and progestins vary in potency and route of administration.^62^ Androgen replacement, such as transdermal testosterone administration, may be considered for persistent fatigue and loss of libido despite optimized estrogen replacement.^63^ HRT should be continued until natural menopause, and then the dose may be tapered to postmenopausal levels or stopped based on individual risks and needs. Clinicians should be knowledgeable about diagnosing and treating POF to prevent unnecessary health risks such as breast cancer, endometrial hyperplasia, thrombotic stroke, and venous thromboembolism for affected women later in life.^64^

## In vitro activation

**Figure 1:**
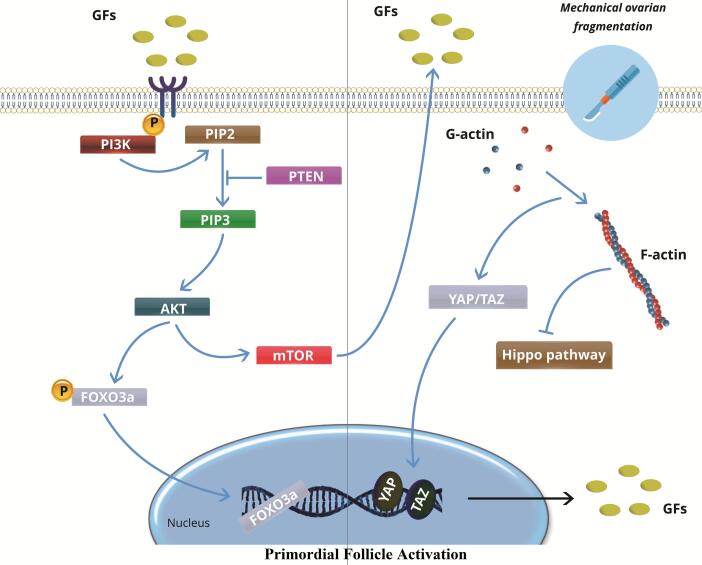
In vitro activation in primary ovarian failure. While PTEN keeps follicles dormant, ovarian cortex fragmentation upsets the Hippo signaling pathway with polymerization of globular actin (G-actin) to filamentous actin (F-actin), dephosphorylating YAP and TAZ to stimulate the transcription of growth factors (GFs), which in turn increases PI3K activity. Activation of the PI3K complex activates PIP2 to PIP3, which results in increased Akt expression. mTOR is upregulated by phosphorylated Akt, which causes primordial follicle activation. In order to encourage the activation of primordial follicles, activated Akt also dephosphorylates and inhibits the functions of FOXO3a. FOXO3: Fork head box O3, Akt: Protein kinase B, mTOR: mammalian target of rapamycin, PI3P: phosphatidylinositol (3,4,5)-trisphosphate, PI2P: phosphatidylinositol 4,5-bisphosphate, PI3K: phosphoinositide 3-kinase, RTK: Receptor tyrosine kinase, PTEN: Phosphatase and tensin homolog, GFs: growth factors, YAP: yes-associated protein, TAZ: transcriptional coactivator with PDZ-binding motif

 One novel strategy for reviving the dormant primordial follicles found in ovaries affected by POF is the concept of IVA. This theory has been supported by recent developments in our knowledge of the function the Hippo signaling pathway plays in triggering these latent follicles, and it may have important therapeutic ramifications for POF patients.^65^ Although there have been worries about the premature activation and depletion of the follicle reserve, dormant follicles have been stimulated by disrupting the Hippo pathway through physically breaking up the ovaries. While a decrease in the number of follicles was observed post-grafting, research using mouse ovaries has shown that fragmenting the tissue and then reinserting it resulted in increased expression of key Hippo signaling genes and a higher proportion of late secondary and antral follicles.^66^ Similarly, studies using Akt-stimulating drug-treated damaged secondary mouse follicles also showed increases in follicle numbers. Additional studies have shown that AKT regulates the activation of primordial follicles, which is mediated by YAP, a crucial downstream effector in the Hippo signaling pathway.^67^ In human experiments, tissue strips were transplanted into immune-deficient mice after human ovarian tissue cubes were fragmented and Akt activation was applied. This resulted in the development of antral follicles. Following ovarian tissue collection and fragmentation, two days of in vitro Akt therapy, and autotransplantation beneath the Fallopian tube serosa, 27 POF patients underwent this procedure. Five patients had mature oocytes extracted, and one patient gave birth to a live child. Eight patients demonstrated follicular growth.^68^ One could reasonably theorize that the positive effects of injecting PRP into the ovaries on ovarian function may result from changes in the Hippo pathway caused by the physical disruption of the ovarian tissue during the injection, rather than from the intraovarian growth factors as previously suggested. This speculation is based on the knowledge of the Hippo signaling pathway and its importance in the development of ovarian follicles (Figure 1).^69^

## Mitochondrial activation

**Figure 2:**
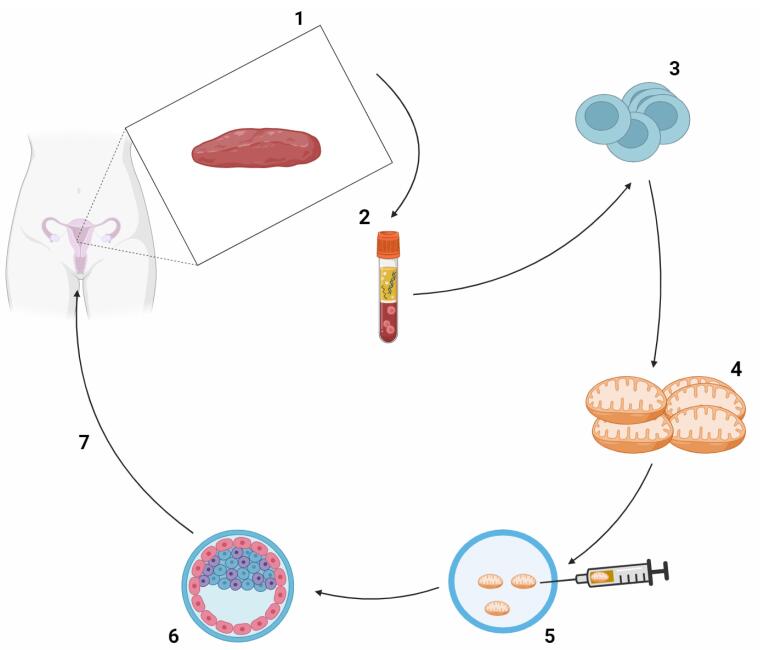
The AUGMENT technique's individual steps are schematically drawn. 1. Under general anesthesia, cortical ovarian tissue is obtained by laparoscopic ovarian biopsy. 2. The obtained ovarian tissue, which contains a mixture of different cell types, is analyzed to select the DDX4-positive oocyte PC population from a cell suspension. 3. The oocyte precursor cells are pelleted by centrifugation to release mitochondria. 4. The patient's released mitochondria are used to prepare a suspension. 5. This suspension is injected alongside the spermatozoon during ICSI. 6. The zygote is cultured in vitro until the blastocyst stage. 7. A good quality embryo is selected for embryo transfer. DDX4, also known as DEAD-box helicase 4, is a protein that plays a crucial role in germ cell development, particularly in the development of oocytes (immature eggs) in females. DDX4-positive oocyte PC likely refers to the presence of DDX4 protein in primordial germ cells (PGCs) or oocytes

 Researchers have been focusing more and more on using pharmacological methods to restore mitochondrial activity in recent years. Coenzyme Q10 (CoQ10), resveratrol, melatonin, and rapamycin are among the nutrients that are accessible for mitochondria. It has been demonstrated that CoQ10, an element of the mitochondrial electron transport chain and a cellular antioxidant, lowers ovarian atresia, increases mitochondrial activity, and restores oocyte mitochondrial gene expression. Renowned for its anti-aging characteristics, resveratrol has been shown to slow down the aging process of ovaries and promote oocyte maturation in vitro. However, because of its anti-deciduogenic activity, which may lower the clinical pregnancy rate, routine clinical use is not advised.^70,71^ It is well known that melatonin has antioxidant qualities that target mitochondria. According to a published study, melatonin can prevent telomerase activity, increase antioxidant capacity, and activate sirtuin1 to postpone ovarian aging.^72^ Additionally, it has been discovered that rapamycin promotes mitochondrial rejuvenation and autophagy. It has been shown to suppress the cyclophosphamide-induced hyperactivation of the PI3K–Akt–mTOR signaling pathway, protecting the ovarian reserve from developing into POF and halting the activation of primordial follicles.^73^ The treatment of infertility with various mitochondrial transfer methods has been investigated. Research has demonstrated that allogeneic ooplasmic transfer in human oocytes can successfully produce pregnancy and a live baby.^74^ However, because of the hazards associated with heteroplasmy, the possibility of mitochondrial disorders spreading, and the likelihood of autism and Turner syndrome following transplantation, this practice has been discontinued.^75^ Pronuclear transfer (PNT), spindle transfer, and germinal vesicle (GV) transfer are examples of novel nuclear transplantation techniques that have been proposed in reaction to these discoveries.^76^ In a spindle transfer procedure, the spindle is removed and placed inside an oocyte donor that has been enucleated.^77^ A 2016 study documented the effective use of this procedure to deliver a kid for a lady with Leigh’s Syndrome, a rare mitochondrial disorder.^77^ GV transfer has been utilized to treat aneuploidy in infertile women by improving meiotic resumption and oocyte maturation.^78^ It’s crucial to remember, though, that mitochondria close to the GV might be transferred into oocytes that have been rebuilt, resulting in mitochondrial heterogeneity that might have a deleterious effect on progeny. Consequently, it is imperative that all mitochondria be removed from patients, even though this is still difficult with GV transfer.^79^ In conclusion, even if mitochondrial transfer treatments appear to be a promising treatment for infertility, one must carefully weigh the hazards involved. Pronuclei from one zygote with aberrant mitochondrial DNA (mtDNA) are transferred to another zygote with normal mtDNA through the process of PNT.^80^ Ethical concerns about the possible loss of zygotes during the process limit the use of PNT.^81^ Autonomous germline mitochondrial energy transfer (AUGMENT) was proposed as a solution to these issues. During intracytoplasmic sperm injection, AUGMENT involves the extraction and transport of mitochondria from oogonial stem cells into the oocyte.^82^ Despite the fact that Oktay et al reported high rates of fertilization and embryonic scoring in women receiving AUGMENT therapy, there are insufficient conclusive clinical trials to support the technique’s efficacy.^83^ Significantly, a recent randomized controlled research raised concerns about the long-term efficacy of AUGMENT therapy by finding that mothers who received it had children with lower mtDNA content than the control group (Figure 2).^84^

## Stem cell therapy

 For those suffering from polycystic ovarian syndrome, stem cell therapy may be able to restore ovarian function and fertility. Early, undifferentiated cells called stem cells have the amazing capacity to self-renew, multiply endlessly, and specialize into a variety of cell types.^85^ Based on where they came from, they are classified as adult stem cells (ASCs), induced pluripotent stem cells (iPSCs), and embryonic stem cells (ESCs). A particular kind of ASC called mesenchymal stem cells (MSCs) is obtained from bone marrow, adipose tissue, menstrual blood, the umbilical cord, amniotic fluid, and the placenta.^86^ Homing, differentiation, and paracrine stimulation are the mechanisms by which stem cells exert their therapeutic effects. Physiologically active molecules, such as growth factors, cytokines, regulatory factors, and signal peptides, are released during paracrine signaling and can have a positive effect on adjacent cells. This process promotes the anti-apoptotic, anti-fibrotic, angiogenic, anti-inflammatory, and immune-regulatory processes that aid in the healing of damaged ovaries.^87^ Despite the great potential for differentiation that ESCs show, the difficult ethical issues surrounding the destruction of blastocysts prevent ESCs from being used in clinical settings. On the other hand, stem cells from human somatic tissues, or iPSCs, present a viable path toward therapeutic application devoid of moral dilemmas. Of specific note, research has shown that human iPSCs can differentiate in vitro into human primitive germ-like cells (hPGCLCs), and under certain conditions, these cells can further differentiate into oogonia/gonocyte-like cells.^88^ Research has indicated that the administration of stem cells, namely bone marrow stem cell (BMSC) injection in mice, can lower free stromal hormone (FSH) levels and raise estrogen and follicle counts via promoting the release of vascular endothelial growth factor (VEGF). Furthermore, it has been discovered that BMSCs prevent granulosa cell death by controlling the expression of particular genes.^89^ Adipose-derived stem cells (ADSCs) present an attractive therapeutic option due to their pluripotency and ease of accessibility. Studies have indicated that the implantation of ADSC triggers angiogenesis, replenishes the corpus luteum and follicle count, and facilitates the restoration of ovarian damage.^90^ Despite ethical debates, MSCs produced from menstrual blood are becoming more and more popular because of their low immunological rejection and toxicity. According to earlier research, MSCs improve POF by preventing granulosa cells from dying and by increasing the release of fibroblast growth factor 2.^91^ Mesenchymal stem cells from the umbilical cord (UC-MSCs) are commonly employed in the management of post-ovarian failure (POF) and have demonstrated the ability to prevent ovarian fibrosis in POF rats by controlling the differentiation of ovarian stromal cells via the transforming growth factor-β/Smad3 signaling pathway.^92^ In vitro, human amniotic fluid stem cells (AFSCs) and amnion mesenchymal stem cells (AMSCs) have both demonstrated potential for growing into primordial follicle oocytes. Furthermore, it has been found that in rats with POF, AMSCs reduce the expression of inflammatory cytokines, which reduces ovarian inflammation. Notably, pretreatment of AMSCs with low-intensity pulsed ultrasound has been shown to enhance their anti-inflammatory capabilities.^93^ In the context of potential risks associated with stem cell therapy, tumorigenicity emerges as a primary concern. Allogeneic transplantation introduces the risk of immune rejection and gives rise to ethical considerations. Furthermore, the elevated cost of these procedures constitutes a significant obstacle to their widespread clinical implementation. To mitigate these concerns, it is imperative to subject stem cell therapy to additional validation through meticulously planned, controlled, and extended clinical trials. These trials play a crucial role in establishing the safety and effectiveness of stem cell therapy across a range of clinical applications.^94^

## PRP Therapy for the Treatment of POF

 Treating POF offers new issues that can be addressed using the innovative technique of intra-ovarian PRP infusion. PRP is made by centrifuging patient peripheral blood, which contains large amounts of platelets. The presence of α-granules is what determines how effective PRP is.^95^ Significantly more proteins, hormones, and growth factors are present in these granules, and when these are released, they promote cell division and proliferation. Moreover, significant amounts of hormones and growth factors are released by active platelets, which support anabolism, angiogenesis, and inflammation control. These processes enable tissues to mend and regenerate quickly. Notably, GDF-9, which is discovered to be mutated in POF patients and is implicated in oocyte maturation, is present in PRP.^96^ PRP has been shown in numerous trials to stimulate primitive and primary follicle development into the presinus stage.^97^ Because of their correlation with preantral and antral follicles and relative stability during the menstrual cycle, AMH levels have been utilized as a primary marker for assessing the effectiveness of PRP.^98^ Furthermore, PRP has demonstrated efficacy in speeding up angiogenesis and decreasing ovotoxic chemical-induced atresia and degeneration in normal follicles.^99^ Pantos et al,^100^ were the first to use PRP in human ovaries for clinical purposes. They administered intraovarian PRP injections to eight perimenopausal women. The study’s findings demonstrated that all patients’ menstrual periods and oocyte retrieval recovered after IVF treatment. PRP has also been used to boost the vascular density of grafts during autologous ovarian transplants due to its beneficial effects on ovarian vascularization.^101^ Studies have shown that after getting PRP treatment, patients with POF were able to conceive naturally through IVF cycles. In every participant studied, these results were linked to decreased levels of luteinizing hormone (LH) and follicle-stimulating hormone (FSH), as well as raised levels of AMH and antral follicle count (AFC).^96^ Furthermore, 23 individuals (7.4%) experienced spontaneous conception following PRP treatment, according to a study assessing the impact of the therapy on ovarian reserves and IVF outcomes in 311 patients with POF.^102^ Chao-Chin et al. coupled PRP with gonadotropin injections into the ovarian stroma to improve the clinical outcome. For a patient who had previously responded badly to gonadotropins, this therapeutic method led to the successful resumption of menstrual cycles and pregnancy.^69^ Autologous PRP intraovarian injection has several benefits, such as low immunogenicity, good storage qualities, and ease of handling. On the other hand, severe cell proliferation events, infection, and unidentified harmful effects on the embryo are possible hazards connected with PRP therapy.^103^ The discovery that certain PRP samples tested positive for microbial growth highlights how crucial it is to identify and neutralize blood-borne infections in samples. Although PRP preparations have demonstrated antibacterial qualities, high-intensity cell proliferation events have the potential to cause cancer because they differentiate stem cells inside the ovaries.^104^ Furthermore, the high concentrations of hematopoietic cells that PRP introduces into the implantation environment run the danger of having a deleterious effect on the embryo.^105^ As a result, more research is required to present conclusive proof of PRP therapy’s safety.

## Exosome Therapy

**Figure 3:**
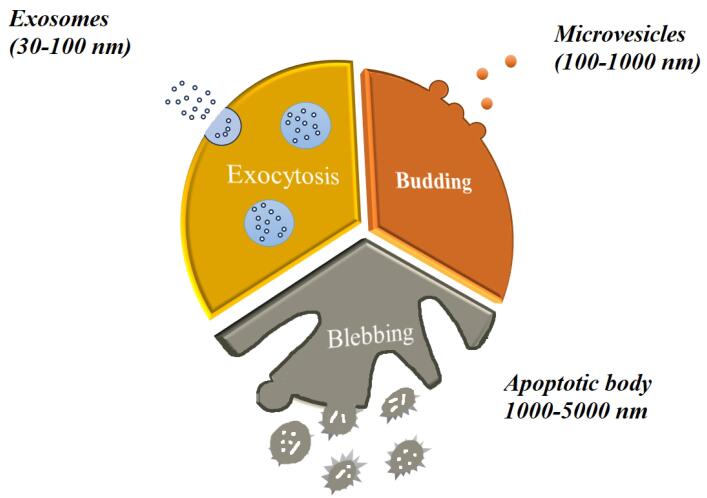
Classification of extracellular vesicles (EVs): three distinct classes of EVs are illustrated. Exosomes: produced by the endocytic pathway, they are released through exocytosis and have a diameter ranging from 30 to 100 nm. Microvesicles (MVs): released by budding from the plasma membrane, they have an irregular shape and a diameter ranging from 100 to 1000 nm. Apoptotic bodies: released through blebbing by cells going through apoptosis, they are larger than 1000 nm in size

 Exosomes, also known as Exos, are a subclass of EVs, a broad category of nanoscale structures released by different cell types in a way that is conserved in all living things. EVs have the ability to transfer genetic information and molecular signals between donor and recipient cells, which can affect the bioactivity and responsiveness of the cells.^106^ EVs are classified as exosomes, micro-vesicles (MVs), and apoptotic bodies (Abs) according to their size, composition, and mode of biogenesis. The largest EVs, known as apoptotic bodies, are formed during apoptotic changes through cellular breakdown. They have an irregular structure and range in diameter from 1000 to 5000 nm.^107^ They consist of various cell components, such as nuclear fragments and organelles. Microvesicles are secreted from both healthy and injured cells in response to a range of stimuli and insults. They can range in size from 100 to 1000 nm. They consist of exovesicles, shedding vesicles, microparticles, and ectosomes. The endosomal-origin exosomes, which range in size from 30 to 100 nm, are isolated by sucrose gradient centrifugation (1.12–1.19 g/mL)^108^ (Figure 3). Different cell types and extracellular fluids in the body, including immune cells, BMSCs, MSCs, breast cancer cells, platelets, and synovial fluid, secrete these specialized double-lipid membrane nanovesicles. These nanovesicles can transfer biologically active molecules like proteins, lipids, growth factors, and carbohydrates to target cells and control their function. When Pan and associates found that released microvesicles externalize receptors during erythrocyte formation in 1983, the word “exosome” was first used.^109^ Exosomes have drawn more interest in the last 10 years because of their potential for both diagnosis and treatment in a number of illnesses.^110^ The formation of intracellular MVBs containing intraluminal vesicles (ILVs) and the stimulation of MVBs to fuse with the plasma membrane in order to release their cargo into the extracellular environment or be degraded by lysosomal activity are the three main steps involved in the formation and release of exosomes. The first step involves the generation of early endosomes (endocytic vesicles) from the cell membrane^110^. Because exosomes are derived from endosomes, they contain a variety of proteins; these include heat shock proteins (Hsp60, Hsp70, Hsp90), tetraspanins (CD9, CD63, CD81, CD82), membrane transport and fusion proteins (GTPases, Annexins, flotillin), and lipids with structural elements like sphingophospholipids, sphingolipids, cholesterol, ceramide, and saccharide groups. Exosome-specific proteins can act as distinguishing indicators for exosome identification^111,112^ (Table 3). Exosomes are produced by the majority of cells and found in biofluids like blood, saliva, urine, amniotic fluid, follicular fluid, uterine fluid, cerebrospinal fluid, ascites fluid, bronchoalveolar lavage, and all types of cells in different in vitro culture systems. They are widely considered as powerful paracrine and autocrine mediators in cellular communication.^113^ Numerous functions, such as cell division and apoptosis, cellular upkeep, metabolism, translational activity, angiogenesis, tissue repair, immune modulation, immune cell processing of antigens, blood coagulation, reproduction, tumor pathogenesis, inflammation spread, autoimmune diseases, neurodegenerative diseases, and infectious diseases have all been shown to be impacted by exosomes. Exosomes have the potential to be used as revolutionary therapeutic bio-shuttle drug delivery systems because of their potential as diagnostic indicators in diseased circumstances.^114-116^ More than 75% of the EVs in the blood are PLT-Exos, the main subtype of EVs released by platelets^12^. PLT-Exos treatment has been demonstrated in rodent models of POF to improve fertility rates by shortening the time to conception, reestablish the estrous cycle, raise hormone and AMH levels, increase follicle count, and prevent follicular atresia.^17^ Studies conducted in vitro have shown that the primary mechanism of ovarian rejuvenation brought about by exosome therapy takes place at the granulosa cell level, which is important in the pathophysiology of post-ovarian fibromatosis. This mechanism is linked to upregulated B-cell lymphoma 2 (Bcl2) and phosphoinositide 3 kinase–protein kinase B (PI3K/Akt) signaling pathways, along with downregulated SMAD and Bcl-2 associated X protein (Bax) signaling pathways. The net effect is increased proliferation and decreased apoptosis.^117^ Additionally, it has been demonstrated that a number of microRNAs included in PLT-Exos, including miR-144-5p, miR-126-3p, and miR-369-3p, suppress granulosa cell death via diverse methods.^118^ Apart from the impact of exosome therapies on granulosa cells, it has also been observed that the transplantation of UC-MSCs in POF rats attenuates ovarian tissue fibrosis and enhances the differentiation of theca cells. This helps to restore the function of the ovaries. Exosome therapy is now recognized by many writers and researchers as a promising therapeutic option for POF because of the encouraging study data that is currently accessible.^119^ Exosomes have been shown to have lower immunogenicity, less tumorigenicity, and no ethical problems when compared to stem cells and PRP. However, their safety and efficacy must be evaluated in human clinical studies before being considered in POF patients (Table 4).^120^

**Table 3:** Characteristics of extracellular vesicles

**Specification**	**Exosome**	**Microvesicles**	**Apoptotic bodies**	**Reference**
Size	30-100 nm	100-1000	1000-5000	^107,108^
Intracellular origin	Endosomal pathways (MVBs)	Plasma membrane	Plasma membrane during apoptosis	^107^
Appearance in electron microscopy	Round shaped	Heterogeneous	Heterogeneous	^108^
Sucrose gradient	1.12-1.19 g/mL	1.04-1.07 g/mL	1.16-1.28 g/mL	^108^
Markers	Tetraspanins (CD9, CD63, CD81, CD82), TSG101, Alix, flotillin, heat shock proteins (Hspa8, Hspa60, Hspa70, Hspa90), ESCRT components, MHC-I and MHC-II molecules	Non-specific markers; Integrins, selectins, CD40, and Annexin V positivity used must: ARF6, VCAMP3	High PS level TSP C3b Annexin V-positivity	^109-111^
Internal contents	mRNA, miRNA, ncRNA, tRNA, siRNA and rarely DNA, Cytoplasmic and membrane proteins (actin, myosin, and syntenin and …), lipids (phosphoglycerides, cholesterol, ceramide, sphingomyelin, and fatty acid chains), carrier proteins like albumin metabolic enzymes (GAPDH, LDHA, PGK1, PKM, PK, ATPase, Enolase and aldolase)	mRNA, miRNA, ncRNA, cytoplasmic and membrane proteins, and thermal shock proteins	Cellular organelles and nuclear fragments	^110,112^
Detection method	Flow-cytometry, electron microscopy, Nanoparticle tracking analysis, Western blotting assay	Flow-cytometry and electron microscopy	Flow-cytometry and electron microscopy	^109^

**Table 4:** Exosome markers, compositions and role of components

**Category**	**Examples**	**Role**	**References**
Tetraspanins	CD9, CD63, CD37, CD81, CD82, CD53	Exosome biogenesis, exosome cargo selection, targeting and uptake	^107-111^
ESCRT machinery/MVB biogenesis	Alix, TSG-101	Exosome biogenesis	^108-110^
Heat Shock Proteins	Hsp90, Hsc70, Hsp60, Hsp20, Hsp27	Exosomes release, signaling	^111,112^
Membrane transport and fusion	GTPases, annexins, flotillin, Rab GTPases, dynamin, syntaxin	Exosome secretion and uptake	^110-112^
Major histocompatibility complex (MHC) molecules	MHC Class I, MHC Class II	Antigen presentation to generate immunological response	^110-112^
Cytoskeletal proteins	Actin, cofilin, tubulin	Exosome biogenesis and secretion	^113-116^
Adhesion molecules	Integrin α, β, P-selectin	Exosome targeting and uptake	^117,118^
Glycoproteins	β-Galactosidase, O-linked glycans, N-linked glycans	Exosomes targeting and uptake	^118^
Growth factors and cytokine	TNF-α, TGF-β, TNF-related apoptosis inducing ligand (TRAIL)	Exosome targeting and uptake, signaling	^117-119^
Other signaling receptors	Fas ligand (FasL), TNF receptor, transferrin receptor (TfR)	Exosome targeting and signaling including apoptosis induction and iron transport	^118-120^

## Conclusion

 A condition with a diverse origin, POF affects up to 3.7% of all females worldwide. Although infertility is seen to be the most crippling aspect of the illness, HRT can aid with its symptomatology and long-term health implications. The reinterpretation of ovarian reserve as a dynamic, rather than static, cell population has prompted research into novel biological strategies for ovarian rejuvenation, including PRP, exosome therapy, IVA, stem cell therapy, microRNAs, and mitochondrial targeting therapies. Intraovarian PRP administration is one of these experimental techniques that has been well explored, is less invasive, and has demonstrated encouraging efficacy, especially in inducing spontaneous conception (7.4%–10%). Furthermore, it has been demonstrated that PRP and IVA are effective in generating conception through in vitro fertilization (IVF), with success rates of 4% and 7%–8%, respectively. However, there hasn’t been much research done on IVA. Given that POF patients are not suitable candidates for IVF therapy using their own oocytes, these techniques may provide them hope for genetically related offspring. Compared with the expensive stem cell-based therapies, PRP and IVA treatments are less expensive. Statistics from the literature on POF-afflicted women show that stem cell transplantation has a limited success rate (0–14.3%, total of 2 cases) in helping women become pregnant. Autologous mitochondrial replacement therapy has not been investigated and has not shown any benefit in patients with low oocyte quality. In preclinical models, emerging biological methods like exosomes and microRNAs show promise in changing disease, but they still need to be further validated in clinical trials. To sum up, biological treatments for POF have potential but are still in the early stages of research. The limited duration of follow-up research on clinically used treatments, such PRP, IVA, and stem cell transplantation therapy, prevents us from making any firm conclusions on the safety or duration of ovarian rejuvenation resulting from these methods. Because there are no standardized procedures for the manufacture and delivery of PRP, it is difficult to compare trials. Furthermore, it is challenging to create standardized guidelines for the choice of stem cell types or the necessary interval between stem cell transplantations due to the dearth of data on the procedure. Consequently, to evaluate the effectiveness, safety, and repeatability of these processes, carefully planned, supervised clinical studies are required. Clinical fertility indices, such as the number of take-home kids and the attainment of clinical pregnancy, should be used to determine success. However, ovarian reserve indices, such as AMH and AFC, may also yield useful information. Even though it is ideal, the isolated increase in hormone levels cannot demonstrate the efficacy of these therapies because standard POF treatment with HRT can yield the same results. Eight clinical trials using PRP application, three involving IVA, and five involving stem cell transplantation are now underway, and the results should be known within the next two years, according to ClinicalTrials.gov. Meticulous experimental and clinical designs will shed light on the safety and effectiveness of these novel therapies for infertility in POF.

## Acknowledgments

 The authors of this research would like to express their gratitude to their colleagues, particularly Dr. Yashar Esmaeilian from the Research Center for Translational Drug at KOÇ University in Istanbul, Turkey. We would also like to acknowledge the laboratory personnel of the Faculty of Advanced Medical Sciences for their assistance.

## Competing Interests

 The research was carried out without any financial or commercial ties that might be seen as having a conflict of interest, the authors disclose.

## Ethical Approval

 This study was approved by the Ethical Committee of Tabriz University of Medical Sciences, Tabriz. IRCT code: (IR.TBZMED.REC.1398.079)
